# Biometric recognition through gait analysis

**DOI:** 10.1038/s41598-022-18806-4

**Published:** 2022-08-25

**Authors:** Claudia Álvarez-Aparicio, Ángel Manuel Guerrero-Higueras, Miguel Ángel González-Santamarta, Adrián Campazas-Vega, Vicente Matellán, Camino Fernández-Llamas

**Affiliations:** Department of Mechanical, Computer Science and Aerospace Engineering, University of León, 24071 León, Spain

**Keywords:** Engineering, Mathematics and computing

## Abstract

The use of people recognition techniques has become critical in some areas. For instance, social or assistive robots carry out collaborative tasks in the robotics field. A robot must know who to work with to deal with such tasks. Using biometric patterns may replace identification cards or codes on access control to critical infrastructures. The usage of Red Green Blue Depth (RGBD) cameras is ubiquitous to solve people recognition. However, this sensor has some constraints, such as they demand high computational capabilities, require the users to face the sensor, or do not regard users’ privacy. Furthermore, in the COVID-19 pandemic, masks hide a significant portion of the face. In this work, we present BRITTANY, a biometric recognition tool through gait analysis using Laser Imaging Detection and Ranging (LIDAR) data and a Convolutional Neural Network (CNN). A Proof of Concept (PoC) has been carried out in an indoor environment with five users to evaluate BRITTANY. A new CNN architecture is presented, allowing the classification of aggregated occupancy maps that represent the people’s gait. This new architecture has been compared with LeNet-5 and AlexNet through the same datasets. The final system reports an accuracy of 88%.

## Introduction

User identification has become increasingly important in different research areas. For example, in the field of cybersecurity, to prevent access to critical facilities or in social and assistive robotics to improve Human-Robot Interaction (HRI).

In the cybersecurity field, it is necessary to take into account that the number of cyber-attacks in different environments has increased exponentially in recent years^1,2^. On access control to critical infrastructures, cybercriminals have many techniques to gain access to facilities either remotely, by obtaining credentials, exploiting vulnerabilities in some of the systems, or physically, by breaching access to the facilities^3,4^. If the access is done remotely, they can be detected in the system when they start to perform malicious actions such as privilege escalation or lateral movements. These techniques are detected by analysing network traffic, system logs, or the system’s overall behaviour. Nevertheless, if the access is physical, an attack named tailgating^5^, a cybercriminal only could be detected by building employees or security personnel if it exists. Thus, more and more companies nowadays implement biometric systems in their infrastructure. The standard Radio Frequency Identification (RFID) cards or passcodes have become outdated.

In robotics, the tasks that a mobile robot has to accomplish are becoming more complex, specifically if we focus on social or assistive robotics. These complex tasks are usually divided into little skills that the robot can solve. First, a robot needs to know its location in the environment where it operates^6^—this is known as localization. Then, it needs to calculate the best path avoiding obstacles or damaging people or objects in their trajectory^7^—aka navigation. Finally, the robot interacts with people and sometimes works with them on specific tasks^8^—aka Human-Robot Interaction (HRI). There are robust solutions for the first two skills because these areas have been extensively studied in the literature. The third one is probably the most complex skill, so many researchers are currently working on it. Human-Robot Interaction (HRI) has to be as similar as possible to human-human interaction^9^. Interaction refers to collision avoidance, but it also involves approaching skills or communication. Such elements are associated with two essential skills: people tracking and recognition. It is necessary to know where the people are every time, but it is also crucial to know who they are.

Tracking people helps to improve navigation skills in mobile robots and promote socially acceptable robots^10^. Many solutions in the literature attempt to solve this problem with Red Green Blue Depth (RGBD) cameras to detect people in the environments as shown in^11^. Other researchers combine data from both Laser Imaging Detection and Ranging (LIDAR) and Red Green Blue Depth (RGBD) cameras^12–14^. However, these approaches have a high computing demand, and it may be a drawback if the tracking runs onboard a robot. Therefore, some solutions have been proposed in the literature to solve the tracking problem using 2D Laser Imaging Detection and Ranging (LIDAR) sensors. For example, the work^15^ reviewed different methods for robot navigation in crowded indoor environments. Furthermore, the clustering and centre point estimation combined with the walking centre line estimation is used to detect people with or without a walker^16^.

The above approaches are not robust enough when dealing with occlusions or changes in gait speed. To address such issues, the authors proposed a Convolutional Neural Network (CNN)-based tool that allows for locating people within the robot surroundings using the data provided by a single Laser Imaging Detection and Ranging (LIDAR) sensor^17^. This tool, called People Tracking (PeTra), creates an occupancy map from the Laser Imaging Detection and Ranging (LIDAR) sensor’s readings. Maps are processed by a Convolutional Neural Network (CNN) which returns segmented data belonging to the people in the robot’s surroundings. A centre-of-mass calculation provides the people’s location estimates from the segmented data. Several versions of the People Tracking (PeTra) have been released. Then, it was included a correlation method of location estimates using Kalman filters, as well as an optimization for the Convolutional Neural Network (CNN)^18^. Finally, a bootstrapping method was proposed to improve the accuracy of the tool in specific locations^19^. People Tracking (PeTra) is the tool selected to detect people in this work.

People recognition is a hot topic. It is not only required in mobile robotics, but also prevalent in our daily life. The use of biometry technologies is very extended. Biometry uses information about a specific part of the human body or behaviour, allowing us to distinguish people by analyzing such data^20^. Biometric technology splits into two main groups. The physiological biometric technologies analyze a specific feature of the body^21^. It is a well-known method since a significant part of society uses it daily. Within this group of techniques, we can find fingerprint^22^, facial^23^, or iris^24^ identification. On the other hand, behavioural biometric technologies analyze actions carried out by people^25^. In this second group, a time component is required since any action has a beginning, developing, and ending^26^. Within this group, we can find voice^27^, hand-writing^28^, or gait identification^29^. The last mentioned work presents a review of the methods used to capture gait information based on vision, sound, pressure, and accelerometry. Gait features can be extracted from a sequence of visual images or video, an underfoot pressure image sequence obtained using a pressure mat sensor, an acceleration trace recorded by an accelerometer in a wearable device, or an audio recording.

As mentioned above, a robot must recognize the person interacting with to promote socially acceptable robots. In robotics, most studies use physiological biometric technologies—specifically face recognition using a camera^30^. However, this sensor has a critical drawback—data processing has a higher computational cost than other sensors. Furthermore, on face recognition, people should face the camera constantly. In addition, the worldwide COVID-19 situation and the consequent masks make this method even more complex. To meet such issues, some authors propose a multi-modal biometric identification combining face and voice identification^31,32^. Finally, it is essential to point out that the use of cameras bounds the users’ privacy^33^. Regarding Laser Imaging Detection and Ranging (LIDAR) sensors, several proposals use these sensors to perform human detection and tracking. This sensor solves the user privacy problem as well as the usability of the system, since the user does not have to constantly look at the robot. Recent studies analyze people’s gait using 2D and 3D Laser Imaging Detection and Ranging (LIDAR) sensors to improve the tracking of individuals.

The work^34^ proposes an Long Short-Term Memory (LSTM)-based method for gait recognition using a multi-line Laser Imaging Detection and Ranging (LIDAR) sensor. The study proposes to create a silhouette from the point cloud extracted from the multi-line LIDAR and process it through an LTSM-based CNN network. The experimental results revealed that the proposed approach achieved a 60% of performance classification for 30 people.

Benedek et al.^35^ presents two approaches, one for gait analysis and the other one for activity analysis, both based on data streams of a Rotating Multi-Beam (RMB) Laser Imaging Detection and Ranging (LIDAR) sensor. The gait analysis is used to do the person re-identification during tracking and recognition of specific activity patterns. They use a silhouette-based approach to projecting the 3D point cloud of a person obtained through the Rotating Multi-Beam (RMB) Laser Imaging Detection and Ranging (LIDAR) sensor to an image 2D plane. This image is preprocessed and evaluated through a Convolutional Neural Network (CNN). The experimental results revealed that the proposed approach achieved an average of 87% of performance classification for 28 people. Finally, a proposal to preserve the user’s privacy, the work^36^, uses a 2D Laser Imaging Detection and Ranging (LIDAR) sensor located at ankle level for people tracking. The study also performed a gait analysis to get person height estimation using Laser Imaging Detection and Ranging (LIDAR) data.

In this work, we present Biometric RecognITion Through gAit aNalYsis (BRITTANY), a tool that allows for identifying people through gait analysis using a 2D Laser Imaging Detection and Ranging (LIDAR) sensor and a Convolutional Neural Network (CNN) to process its data. A 2D Laser Imaging Detection and Ranging (LIDAR) sensor was selected because of its low computational requirements and its benefits for users’ privacy. The gait analysis has been chosen because it is a behavioural biometry method. Physiological biometry-based systems only process data gathered in a specific time instant. On the other hand, behavioural biometry-based strategies collect input data during a time interval. It allows for analysing more than one sample of user data, so they get more robust evidence without using any other external identification source.

The remainder of the paper organises as follows: “Materials and methods” section describes the materials and evaluation methods used to carry out our research; the results are presented and discussed in “Results and discussion” section; finally, conclusions and future works are proposed in “Conclusion” section.

**Figure 1:**
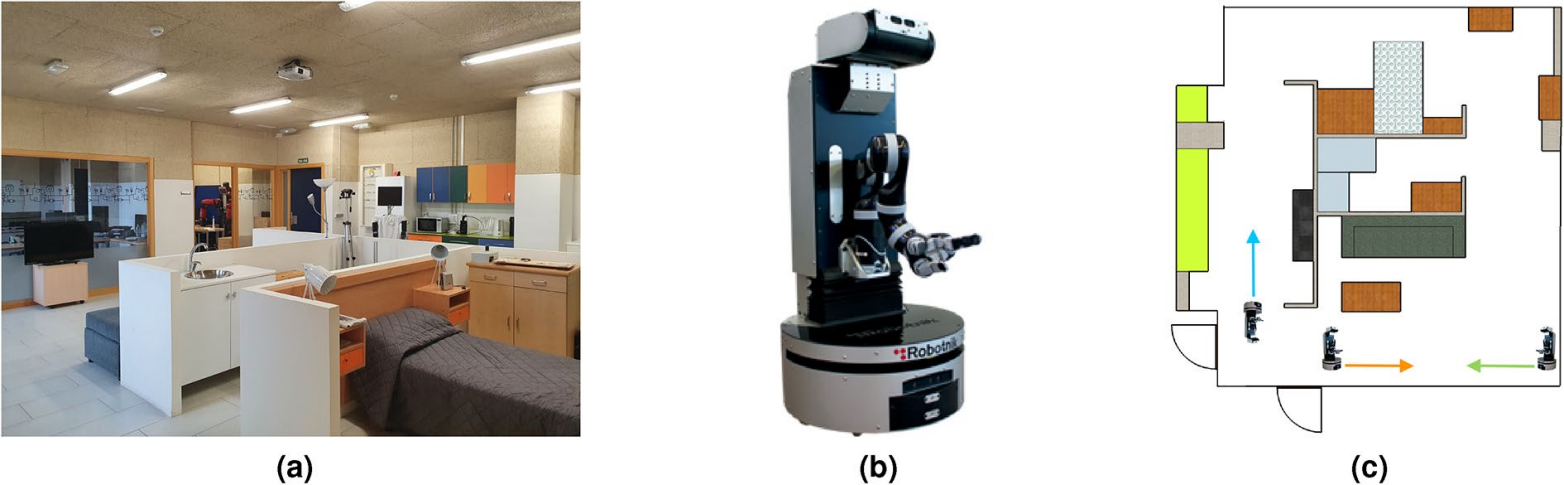
(**a**) Leon@Home Testbed. (**b**) Orbi-One Robot. (**c**) Leon@Home Testbed schema. The robot icons on the map point out the robot’s location and orientation during the experiments; the arrows show the trajectory of people.

## Materials and methods

A set of experiments were carried out to evaluate Biometric RecognITion Through gAit aNalYsis (BRITTANY). In this section, the main elements of the research are in-depth depicted. Besides, we describe the methodology used to assess the accuracy of the proposed system.

### Leon@Testbed

The experiments have been done in the mock-up apartment Leon@Home Testbed^37^, a certified testbed^38^ of the European Robotics League (ERL) located in the Robotics Group’s laboratory at the University of Leon—see Fig. 1a. It is used to test mobile service robots in a realistic environment. The apartment simulates a single-room home built in an 8 m $\times$ 7 m space. 60 cm-high walls—for allowing seeing—split the space into a living room, kitchen, bedroom, and bathroom.

### Orbi-One robot

Orbi-One, the mobile service robot shown in Fig. 1b has been used to gather data. It is manufactured by Robotnik^39^. It accommodates several sensors, such as a Red Green Blue Depth (RGBD) camera, a Laser Imaging Detection and Ranging (LIDAR) sensor, and an inertial unit. It also operates a six-degrees-freedom manipulator arm attached to its torso and a wheeled base for moving around the floor. Inside, an Intel Core i7 CPU with 8 GB of RAM allows it to run the software to control the robot hardware. The software runs the Robot Operating System (ROS) framework^40^. Specifically, we used its onboard Laser Imaging Detection and Ranging (LIDAR) sensor to collect data for our research.

### PeTra

People Tracking (PeTra)^17^ is developed by the Robotics Group at the University of León^41^ in recent years. People Tracking (PeTra) allows for locating people in the environment. It uses data provided by a Laser Imaging Detection and Ranging (LIDAR) sensor accommodated 20cm above the floor. It was evaluated by using an open-access dataset^42^. Starting from Laser Imaging Detection and Ranging (LIDAR) data People Tracking (PeTra) builds a 2D occupancy map that depicts the main features of the robot environment. Such occupancy map is then processed by a Convolutional Neural Network (CNN) that returns a new occupancy map containing only the points that belong to people. People Tracking (PeTra)’s Convolutional Neural Network (CNN) is based on the U-net architecture^43^, commonly used to perform biomedical image segmentation^44^. A centre-mass algorithm is computed over the new occupancy map to estimate the people’s location. New versions of the tool were released in later years. People Tracking (PeTra) can correlate location estimates by using Euclidean distances^45^ or using Kalman filters^46^—a more robust correlation method. An optimized design for the Convolutional Neural Network (CNN) that allows People Tracking (PeTra) for working in real-time^18^. Finally, a bootstrapping-based method that improves the accuracy at specific locations, such as empty rooms or corridors^19^.

### BRITTANY

Biometric RecognITion Through gAit aNalYsis (BRITTANY) allows for recognizing people by their gait. The system is based on a Convolutional Neural Network (CNN) which uses an aggregation of occupancy maps provided by People Tracking (PeTra) as input. We pose that such aggregated occupancy maps are unique for each person and may be used to identify them.

Aggregated occupancy maps are processed to get probability values for each legitimate user. For instance, for five people, Biometric RecognITion Through gAit aNalYsis (BRITTANY) might get [0.01, 0.96, 0.2, 0.24, 0.09] probability values, meaning that input data belongs to the first person with a 0.01 probability, to the second one with a 0.96 probability, to the third one with a 0.2 probability, to the fourth one with a 0.24 probability, and the fifth one with a 0.09 probability. Thus, we might assert that the input data belongs to the second person.

We consider several predictions during a time interval to prevent punctual errors. Thus, we evaluate the final estimation through a set of predictions by applying a most-voted item strategy.

#### Data gathering

Two datasets, available online^47^, have been compiled. First, we gathered data in the mock-up apartment at Leon@Home Testbed described in “Leon@Testbed” section. Data was collected from the Laser Imaging Detection and Ranging (LIDAR) sensor onboard the Orbi-One robot mentioned in “Orbi-One robot” section. Both datasets consist of Rosbag files, a Robot Operating System (ROS) feature that allows for recording data during a time interval and playing them later.

The first dataset ($\mathscr {D}_1$) is composed of 90 five-second Rosbag files. Data recorded correspond to a person walking straight in front of the robot. Each Rosbag file contains the data gathered by the Laser Imaging Detection and Ranging (LIDAR) sensor onboard the robot. We recorded data from five different people at three locations—shown in Fig. 1c on the testbed schema. We recorded six Rosbag files for each location and person.

The second dataset ($\mathscr {D}_2$) is composed of 108 Rosbag files. We recorded data from six people walking straight in front of the robot at three locations—see Fig. 1c. We recorded six Rosbag files for each location and person. Five out of those six people are the same in both datasets. The last one is not registered in the system. We use its data to evaluate the false-positive cases in the system.

#### Data curation

Once we have gait data recorded as Rosbag files, it is necessary to curate and tag them to use them for fitting the Convolutional Neural Network (CNN) in charge of people identification. $\mathscr {D}_1$, depicted in “Data gathering” section, have been used to train the Convolutional Neural Network (CNN).

**Figure 2:**
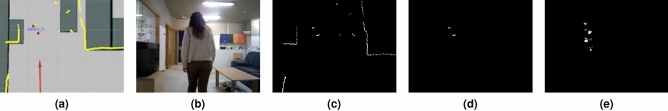
Data curation process: (**a**) Laser Imaging Detection and Ranging (LIDAR) and People Tracking (PeTra) data visualized on Rviz—The red arrow shows the robot’s location an orientation, the yellow points show Laser Imaging Detection and Ranging (LIDAR) readings; (**b**) snapshot from Orbi-One robot camera; (**c**) occupancy map computed from Laser Imaging Detection and Ranging (LIDAR) data; (**d**) occupancy map computed by People Tracking (PeTra); and (**e**) occupancy map aggregation.

Since Biometric RecognITion Through gAit aNalYsis (BRITTANY) uses a Convolutional Neural Network (CNN) to identify people, we need to convert data from Rosbag files into images $\mathscr {I}$ that describe people’s gait. Image generation process is shown in Fig. 2. The Fig. 2a shows the Laser Imaging Detection and Ranging (LIDAR) data as yellow points for the real scene shown in Fig. 2b. The red arrow shows the location and orientation of the robot. First, we play each Rosbag file obtaining occupancy maps for each Laser Imaging Detection and Ranging (LIDAR) reading—see Fig. 2c. From the above occupancy maps, People Tracking (PeTra) provides a second occupancy map segmenting the points belonging to people—see Fig. 2d. Finally, People Tracking (PeTra)’s occupancy maps are aggregated. Such aggregation allows for depicting people’s gait—see Fig. 2e.

A individual occupancy map is represented as an image (*I*), see Fig. 2d. In these images, white pixels represent a Laser Imaging Detection and Ranging (LIDAR) point belonging to a person, and black pixels represent 1) Laser Imaging Detection and Ranging (LIDAR) points belonging to objects, or 2) points where the Laser Imaging Detection and Ranging (LIDAR) sensor has not detected collisions. The final image ($\mathscr {I}$) is created by concatenating individual occupancy maps, see Fig. 2e. These occupancy maps are concatenated using the logical OR (||) operation. Equation (1) represents the operation performed to create the final aggregate occupancy maps. In the Equation, $\mathscr {I}$ represents the final image obtained from the concatenation of individual images (*I*), *n* is the number of *I* used in the aggregation, and *s* is the number of steps between successive images (*I*).

1 $$\begin{aligned} \mathscr {I} = \mathop {||}\limits_{{i = 0}}^{n} I_{{i(s + 1)}} \end{aligned}$$

**Figure 3:**
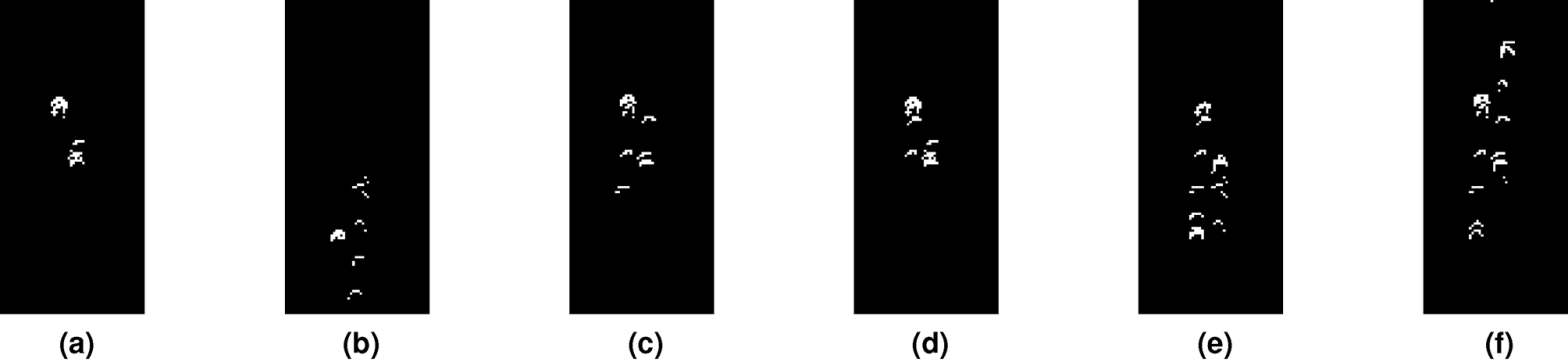
Aggregated occupancy maps for (**a**) $\mathscr {C}_{5 \times 0}$, (**b**) $\mathscr {C}_{5 \times 1}$, (**c**) $\mathscr {C}_{5 \times 2}$, (**d**) $\mathscr {C}_{10 \times 0}$, (**e**) $\mathscr {C}_{10 \times 1}$, and (**f**) $\mathscr {C}_{10 \times 2}$.

Different aggregation settings have been evaluated to select the best one. They are named as $\mathscr {C}_{n \times s}$, where $n \in \langle 5,10 \rangle$ is the number of Laser Imaging Detection and Ranging (LIDAR) readings used in the aggregation, and $s \in \langle 0,1,2 \rangle$ is the number of steps between successive readings. For instance, in $\mathscr {C}_{5 \times 2}$, five Laser Imaging Detection and Ranging (LIDAR) readings are used by considering one out of three successive Laser Imaging Detection and Ranging (LIDAR) readings. Figure 3 show a sample of the resulting aggregated occupancy map for each setting schema.

#### Convolutional neural network design

Convolutional Neural Network (CNN) usage has increased in recent years since a large number of systems integrate them^48^. Convolutional Neural Network (CNN) identify features from tagged datasets. Such features are often imperceptible to humans. Biometric RecognITion Through gAit aNalYsis (BRITTANY) uses a classification Convolutional Neural Network (CNN) to identify people. It receives an occupancy map ($\mathscr {I}$) as input and returns a success rate for each person it was trained. To define the Convolutional Neural Network (CNN), Keras API^49^ have been used using TensorFlow^50^ as back-end.

To carry out the image classification to be performed by BRITTANY, we propose a new neural network architecture, hereafter ”custom”. To evaluate its performance, we have selected two other well-known architectures to perform image classification LeNet-5^51^ and AlexNet^52^. These two architectures have been selected because they are the first well-known architectures that solved image classification problems. Both architectures have been modified to process as input a 256x256 image and as output a Dense layer of 5. Other architectures such as VGG16^53^ have been tested, but due to the complexity in the deep layers, the model created did not generalize correctly to perform the image classification used by Biometric RecognITion Through gAit aNalYsis (BRITTANY).

**Figure 4:**
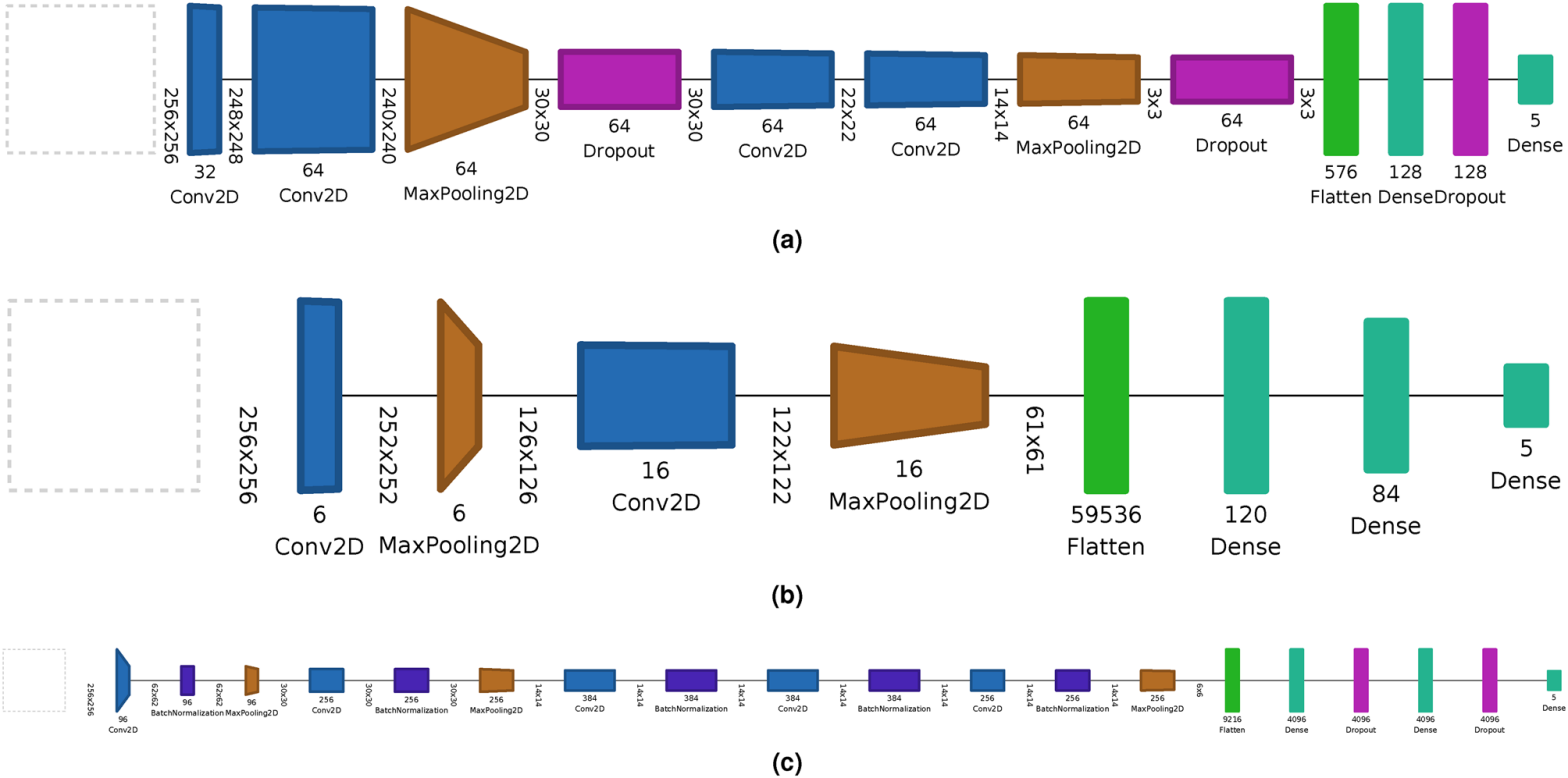
CNN architectures of (**a**) Custom, (**b**) LeNet, (**c**) AlexNet.

Figure 4, generated with Net2vis tool^54^, illustrates the three architectures, Fig. 4a represents the custom architecture proposed in this work, and Fig. 4b and c represents the architecture of LeNet and Alexnet respectively. In addition, Table 1 shows the number of trainable and non-trainable parameters for each proposed architectures. As can be seen, the custom architecture has a much lower number of trainable parameters than the other two well-known models.

**Table 1:** Parameters for each architecture, first column represents the architecture, the rest represents the trainable, non-trainable and total parameters of each architecture.

Architecture	Trainable parameters	Non-trainable parameters	Total parameters
Custom	906,757	0	906,757
LeNet	7,157,601	0	7,157,601
AlexNet	58,281,349	2752	58,284,101

The Convolutional Neural Networks (CNNs) models were trained on Caléndula the parallel computing cluster of Supercomputación Castilla y León (SCAYLE)^55^. Supercomputación Castilla y León (SCAYLE) is a public research centre dependent on the Community of Castilla y León (Spain) whose main activity is to support the improvement of R & D & I tasks. Six Convolutional Neural Network (CNN) models have been created for each architecture, one for each out of 6 aggregation settings ($\mathscr {C}_{5 \times 0}$, $\mathscr {C}_{5 \times 1}$, $\mathscr {C}_{5 \times 2}$, $\mathscr {C}_{10 \times 0}$, $\mathscr {C}_{10 \times 1}$, and $\mathscr {C}_{10 \times 2}$) depicted in “Data curation” section. In this way, a total of 18 Convolutional Neural Network (CNN) models have been trained.

### Evaluation

The evaluation was carried out using the $\mathscr {D}_2$—see “Data gathering” section. We played each Rosbag file using Biometric RecognITion Through gAit aNalYsis (BRITTANY) with different setting schemas to evaluate the accuracy. We need to know whether or not the user has been recognized properly for each run. Such data allow us to obtain the confusion matrix that allows visualization of the performance of our tool with the six setting schemas defined and the three Convolutional Neural Network (CNN) architectures used, described in “Convolutional neural network design” section.

Moreover, to evaluate the overall Biometric RecognITion Through gAit aNalYsis (BRITTANY)’s performance the following Key Performance Indicators (KPI)’s obtained through the confusion matrix are considered: Accuracy ($\mathscr {A}$), Precision ($\mathscr {P}$), Recall ($\mathscr {R}$), and F$_1$-score ($\mathscr {F}$). As the proposed method is a multi-class classification, it is necessary to calculate Key Performance Indicators (KPI)’s for each class. Then an arithmetic average is calculated to obtain the Key Performance Indicators (KPI)’s of the global system^56^ $\mathscr {A}$—see Eq. (6)—measures the proportion of correct predictions, both positive and negative cases, among the total number of cases examined. $\mathscr {A}_{k}$ is calculated as shown Eq. (2). The $\mathscr {P}$ score—see Eq. (7)—shows the fraction of relevant instances among the retrieved instances. $\mathscr {P}_{k}$ is calculated as shown Eq. (3). The $\mathscr {R}$ score—see Eq. (8)—shows the rate of positive cases that were correctly identified by the algorithm. $\mathscr {R}_{k}$ is calculated as shown Eq. (4). Finally, the $\mathscr {F}$ score—see Eq. (9) is the harmonic mean of precision and recall. $\mathscr {F}_{k}$ is calculated as shown Eq. (5). In equations, *TP* represents the true-positive rate, *TN* is the true-negative rate, *FP* is the false-positive rate, *FN* is the false-negative rate and *K* the number of classes into which the model classifies.

2 $$\begin{aligned} \mathscr {A}_{k} = \frac{TP_{k}+TN_{k}}{TP_{k}+FP_{k}+TN_{k}+FN_{k}} \end{aligned}$$

3 $$\begin{aligned} \mathscr {P}_{k} = \frac{TP_{k}}{TP_{k}+FP_{k}} \end{aligned}$$

4 $$\begin{aligned} \mathscr {R}_{k} = \frac{TP_{k}}{TP_{k}+FN_{k}} \end{aligned}$$

5 $$\begin{aligned} \mathscr {F}_{k} = 2 \frac{\mathscr {P}_{k} \times \mathscr {R}_{k}}{\mathscr {P}_{k} + \mathscr {R}_{k}} \end{aligned}$$

6 $$\begin{aligned} \mathscr {A} = \frac{\sum _{k=0}^{K} \mathscr {A}_{k}}{K} \end{aligned}$$

7 $$\begin{aligned} \mathscr {P} = \frac{\sum _{k=0}^{K} \mathscr {P}_{k}}{K} \end{aligned}$$

8 $$\begin{aligned} \mathscr {R} = \frac{\sum _{k=0}^{K} \mathscr {R}_{k}}{K} \end{aligned}$$

9 $$\begin{aligned} \mathscr {F} = \frac{\sum _{k=0}^{K} \mathscr {F}_{k}}{K} \end{aligned}$$

To evaluate the tradeoff between the TP and FP rates of each class, we have computed the Receiver Operating Characteristic (ROC) curve^57^ for each one of the six setting schemas defined and the three proposed architectures. Besides, the Area Under the Curve (AUC) has been calculated to depict how much the models can distinguish between classes.

## Results and discussion

**Figure 5:**
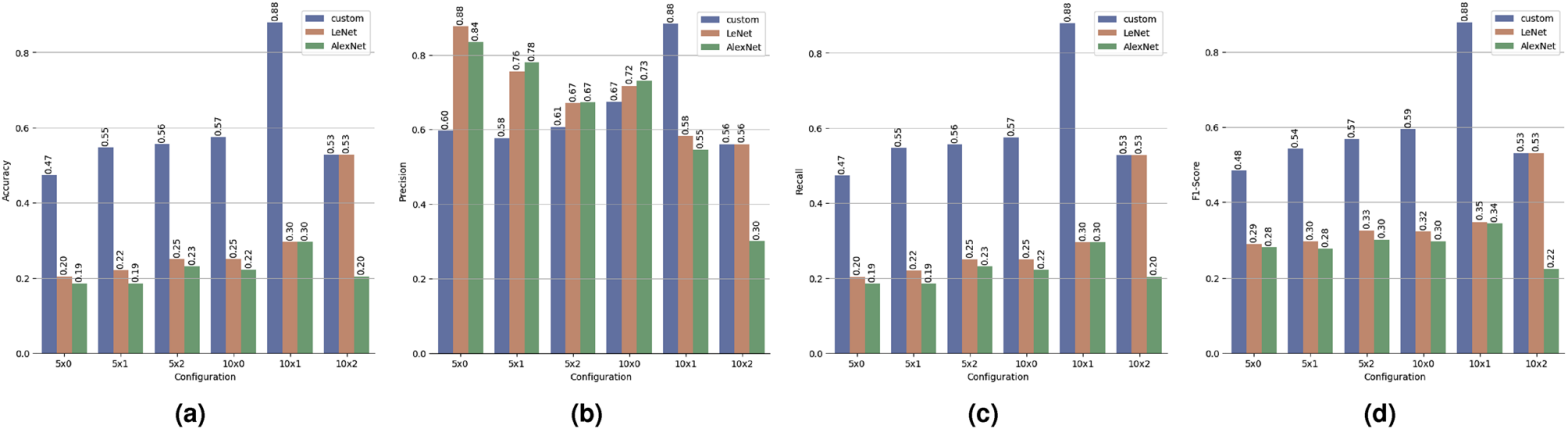
Key Performance Indicators (KPI)’s: Accuracy, Precision, Recall and F1-score, for each of the trained models and each configuration proposed ($\mathscr {C}_{5 \times 0}$, $\mathscr {C}_{5 \times 1}$, $\mathscr {C}_{5 \times 2}$, $\mathscr {C}_{10 \times 0}$, $\mathscr {C}_{10 \times 1}$, and $\mathscr {C}_{10 \times 2}$).

**Figure 6:**
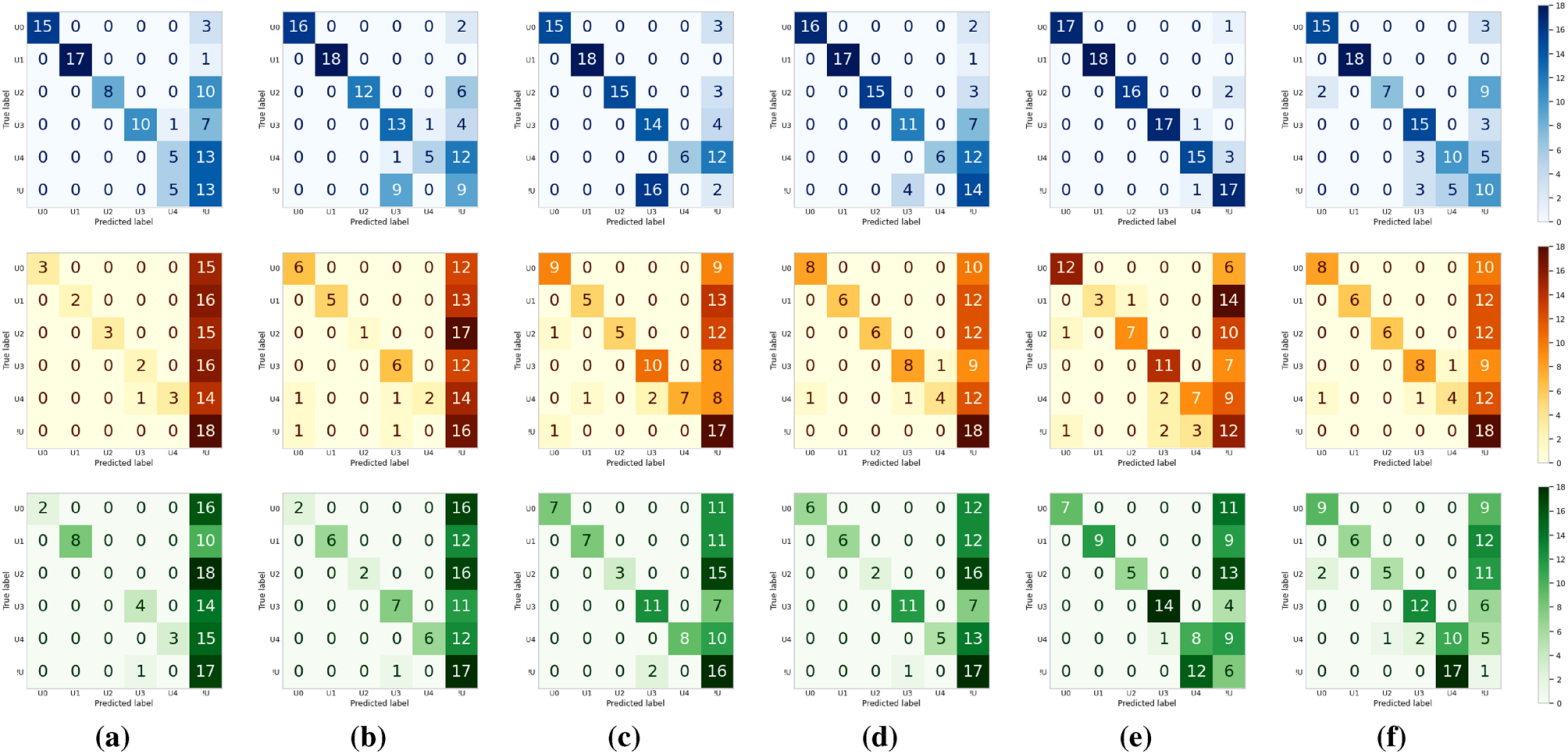
Confusion matrices for each architecture: Custom in blue (first row), LeNet in brown (second row) and AlexNet in green (third row). From left to right the different configurations used: (**a**) $\mathscr {C}_{5 \times 0}$, (**b**) $\mathscr {C}_{5 \times 1}$, (**c**) $\mathscr {C}_{5 \times 2}$, (**d**) $\mathscr {C}_{10 \times 0}$, (**e**) $\mathscr {C}_{10 \times 1}$, and (**f**) $\mathscr {C}_{10 \times 2}$.

**Figure 7:**
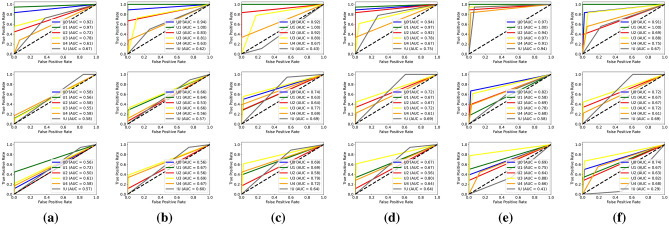
Receiver Operating Characteristic (ROC) curves and Area Under the Curve (AUC) for custom (first row), LeNet (second row) and AlexNet (third row). From left to right the different configurations used: (**a**) $\mathscr {C}_{5 \times 0}$, (**b**) $\mathscr {C}_{5 \times 1}$, (**c**) $\mathscr {C}_{5 \times 2}$, (**d**) $\mathscr {C}_{10 \times 0}$, (**e**) $\mathscr {C}_{10 \times 1}$, and (**f**) $\mathscr {C}_{10 \times 2}$.

Biometric RecognITion Through gAit aNalYsis (BRITTANY)’s evaluation was done as described in “Evaluation” section. We obtained a confusion matrix for each model (custom, LeNet and AlexNet) and each setting schema ($\mathscr {C}_{5 \times 0}$, $\mathscr {C}_{5 \times 1}$, $\mathscr {C}_{5 \times 2}$, $\mathscr {C}_{10 \times 0}$, $\mathscr {C}_{10 \times 1}$, and $\mathscr {C}_{10 \times 2}$). Figure 6, shows the confusion matrices for each model and setting schema. In the first row are the confusion matrices, in blue, for the custom models, in brown (second row) the confusion matrices for LeNet and in the third row, in green, the confusion matrices for AlexNet. From left ($\mathscr {C}_{5 \times 0}$) to right ($\mathscr {C}_{10 \times 2}$) are the different configurations used. Every confusion matrix consists of rows and columns representing user identifiers. The matrices check situations where BRITTANY provided a correct or wrong outcome using each model respectively. A perfect system would have all the values on the main diagonal. According to the values shown in Fig. 6, the Key Performance Indicators (KPI)’s, the Receiver Operating Characteristic (ROC) curve and the Area Under the Curve (AUC) have been calculated for each of the architectures and setting schemas. These Key Performance Indicators (KPI)’s are presented in Fig. 5 that show the Accuracy ($\mathscr {A}$)—Fig. 5a, Precision ($\mathscr {P}$)—Fig. 5b, Recall ($\mathscr {R}$)—Fig. 5c, and F$_1$-score ($\mathscr {F}$)—Fig. 5d, for each model proposed and each setting schema. Moreover in Fig. 7 are presented the Receiver Operating Characteristic (ROC) curves for each model and settting schema. In the first row Receiver Operating Characteristic (ROC) curves for the custom models, in the second row Receiver Operating Characteristic (ROC) curves for LeNet and in the third row Receiver Operating Characteristic (ROC) curves for AlexNet. From left ($\mathscr {C}_{5 \times 0}$) to right ($\mathscr {C}_{10 \times 2}$) are the different configurations used. The Receiver Operating Characteristic (ROC) curves have been created for each user in the system (U0 - U4) and the not registered user (!U), in this way, it is also calculated the Area Under the Curve (AUC) for each user, model and setting schema.

Focusing on Fig. 5a, we see that accuracy scores that measure the proportion of correct (both positive and negative) predictions among the total number of cases examined. The accuracy score is only higher than 0.8, specifically 88%, with the $\mathscr {C}_{10 \times 1}$ schema using the custom model. The accuracy scores in the schemas for the custom model are between 47% and 57%. The accuracy of LeNet and AlexNet for each schema is much lower than that obtained with the custom model, obtaining values between 19% and 30%. Except for the $\mathscr {C}_{10 \times 2}$ schema where LeNet obtains the same value as custom (53%).

The precision ($\mathscr {P}$), see Fig. 5b, represents the fraction of relevant instances among the retrieved samples. In this case, most of the values are higher than 50% for all the models; only the model AlexNet with schema $\mathscr {C}_{10 \times 2}$ reports a precision of 30%. The two models with a better precision are LeNet with the schema $\mathscr {C}_{5 \times 0}$ and custom with the schema $\mathscr {C}_{10 \times 1}$, for both models, the precision score is 88%.

The recall score ($\mathscr {R}$)—also called sensitivity or true positive rate—, see Fig. 5c is the ratio of positive instances correctly detected by the algorithm. In this score, the maximum value, 88%, is again for the custom model and $\mathscr {C}_{10 \times 1}$ schema. The recall scores in the schemas for the custom model are between 47% and 57%. The recall of LeNet and AlexNet for each schema report values between 19% and 53%.

It is often convenient to combine precision and recall into a single metric, see Fig. 5d. The $F_1$ score ($\mathscr {F}$) is the harmonic mean of $\mathscr {P}$ and $\mathscr {R}$. Whereas the regular mean treats all values equally, the harmonic mean gives much more weight to low values. As a result, the classifier will only get a high $F_1$ score if both recall and precision are high. The model with the best $F_1$ score is the custom using the schema $\mathscr {C}_{10 \times 1}$, specifically 88%. The remaining schemas for the custom model have a $F_1$ score between 48% and 59%. The $F_1$ score of LeNet and AlexNet for each schema is much lower than that obtained with the custom model, obtaining values between 22% and 35%. Except for the $\mathscr {C}_{10 \times 2}$ schema where LeNet obtains the same value as custom (53%).

The model with the best Key Performance Indicators (KPI)’s is custom using the schema $\mathscr {C}_{10 \times 1}$, all the Key Performance Indicators (KPI)’s reports a score of 88%. Then, focusing on the confusion matrix of the custom model and the best schema, $\mathscr {C}_{10 \times 1}$, shown in Fig. 6 (first row, fifth column), we see that most of the values are in the main diagonal. However, there are some errors. U1 was the only correctly identified user in all cases—we have 18 cases, six possible users at three different locations. U0 user was recognised as U0 in 17 cases. However, there is one case where U0 was not recognised as a registered user (!U). U2 was identified correctly in 16 cases, and he was not recognised (!U) in 2. U3 was correctly identified in all cases but one, where he was recognised as U4. It is important to point out that this is the only case in the evaluation of the $\mathscr {C}_{10 \times 1}$ schema and the custom model where two registered users were mistaken. The results for U4 are the worst in the evaluation. In 15 cases, the user was correctly identified, but in 3 cases, he was not recognised (!U). Finally, a non-registered user was used to test the Biometric RecognITion Through gAit aNalYsis (BRITTANY)’s robustness in front of unknown users. Such a user was wrongly recognised as U3 in 1 case. In the remaining 17 cases, he was correctly identified as non-registered.

Focusing on the Receiver Operating Characteristic (ROC) curves for each of the models obtained, see Fig. 7. It can be seen that the best representation of the Receiver Operating Characteristic (ROC) curves corresponds to the custom model and the $\mathscr {C}_{10 \times 1}$ schema (first row, fifth column). The quality of the model increases when the curve moves towards the upper left corner of the graph. This is because it improves its *TP* rate, also minimising the *FP* rate. Moreover, the Area Under the Curve (AUC) values are used as a summary of the model’s performance. The more curve moves towards the upper left corner of the graph, the more area is contained under it and therefore, the classifier is better. A perfect classifier has an Area Under the Curve (AUC) of 1. The Area Under the Curve (AUC)’s for each user obtained from BRITTANY using the custom model and the $\mathscr {C}_{10 \times 1}$ schema are (0.97, 1.00, 0.94, 0.97, 0.91 and 0.94) respectively, all of them higher than 90%.

Finally, the accuracy obtained from Biometric RecognITion Through gAit aNalYsis (BRITTANY) using the custom model and the $\mathscr {C}_{10 \times 1}$ schema is 88%. Focussing on works presented in the Introduction that applied biometry technologies, the work^34^ creates a silhouette from the point cloud extracted from the multi-line LIDAR and processes it through an LTSM-based CNN network obtaining an accuracy of 60%. The work^35^ uses gait analysis to do person re-identification, using the silhouette obtained from the projection of the 3D point cloud of a person to a 2D image. This method obtains an accuracy of 87%. It should be noted that those works use a 3D LIDAR instead of a 2D LIDAR. The number of values provided by a 2D LIDAR sensor is much smaller than the ones provided by a 3D LIDAR, so it is easier to process data. This fact facilitates the use of the final system in real-time. Moreover, the models used in those works have not been evaluated against users outside the system. Therefore, people who are not registered in the system could be classified as legitimate users of it.

## Conclusion

This paper presents Biometric RecognITion Through gAit aNalYsis (BRITTANY), a system that identifies people by analyzing their gait. Thus, the system is based on behavioural biometric technologies characterized for analyzing the features of a specific action performed by a person. This system processes sensor data obtained in real-time. A Laser Imaging Detection and Ranging (LIDAR) sensor was chosen because of its low computational demand as well as the privacy it provides. The Laser Imaging Detection and Ranging (LIDAR) readings are processed by People Tracking (PeTra) to create an occupational map segmenting the points that belong to people. Then, such segmented occupational maps are aggregated to build an image of people’s gait. Aggregated occupancy maps are processed by a Convolutional Neural Network (CNN) model that outcomes the user identifier that corresponds to the person in front of the robot. The final prediction depends on several estimations by applying a voting strategy to prevent errors. Biometric RecognITion Through gAit aNalYsis (BRITTANY) can be used in several applications on cooperative robotics and raises Human-Robot Interaction (HRI) since it allows the robot to “know” the people around him. Moreover, at indoor environments, the robot could detect foreigners and alert their presence. Besides, it also can be used to control access to critical infrastructures, making it more difficult for cybercriminals to carry out tailgating attacks.

The evaluation was done by analyzing several setting schemas named $\mathscr {C}_{n \times s}$ with three Convolutional Neural Network (CNN) architectures, one of them is proposed in this work, called ”custom” and the other two are well-known architectures (LeNet and AlexNet). The dataset $\mathscr {D}_2$ was used to measure the performance. It compiles 108 Rosbag files—Six recordings for six users at three different locations. Five users (U0–4) are well-known people. The last one (!U) is unknown to the system and was used to evaluate the system’s performance in front of strange people. The $\mathscr {C}_{10 \times 1}$ schema using the custom model gets the best results in all the Key Performance Indicators (KPI)’s computed. Such schema builds images by aggregating ten segmented occupancy maps taking one out of two. This schema provides an accuracy score of 88%.

As previously mentioned, this work is a Proof of Concept (PoC) for indoor environments with few people. We aim to determine if it is possible to identify people by their gait using a 2D LIDAR sensor to maintain the user’s privacy and reduce computational load. In future work, we propose to collect data from more users to evaluate Biometric RecognITion Through gAit aNalYsis (BRITTANY) as an authentication tool.

We want to point out that all the datasets generated are available online^47^. Besides, The source code developed during the current study is available under an open-source license in the GitHub repository^58^. Finally, a docker image with all required software to double-check the evaluation posed in this paper is also available online^59^.

## Data Availability

The datasets generated and/or analysed during the current study are available in the “Dataset for train and test BRITTANY (Biometric RecognITion Through gAit aNalYsis)” repository, 10.5281/zenodo.5825885.
